# Hypothermia treatment ameliorated cyclin-dependent kinase 5-mediated inflammation in ischemic stroke and improved outcomes in ischemic stroke patients

**DOI:** 10.6061/clinics/2019/e938

**Published:** 2019-10-14

**Authors:** Hongwei Sun, Juan Cai, Shiqi Shen, Xiaohui Ren

**Affiliations:** The First Affiliated Hospital of Harbin Medical University, Harbin 150001, Heilongjiang, China

**Keywords:** Hypothermia, Ischemic Stroke, CDK5, IL-1β, Inflammation

## Abstract

**OBJECTIVES:**

The inflammatory response is a key mechanism of neuronal damage and loss during acute ischemic stroke. Hypothermia has shown promise as a treatment for ischemic stroke. In this study, we investigated the molecular signaling pathways in ischemic stroke after hypothermia treatment.

**METHODS:**

Cyclin-dependent kinase 5 (CDK5) was overexpressed or silenced in cultured cells. Nuclear transcription factor-κB (NF-κB) activity was assessed by measurement of the luciferase reporter gene. An ischemic stroke model was established in Sprague-Dawley (SD) rats using the suture-occluded method. Animals were assigned to three groups: sham operation control, ischemic stroke, and ischemic stroke + hypothermia treatment groups. Interleukin 1β (IL-1β) levels in the culture supernatant and blood samples were assessed by ELISA. Protein expression was measured by Western blotting.

**RESULTS:**

In HEK293 cells and primary cortical neuronal cultures exposed to hypothermia, CDK5 overexpression was associated with increased IL-1β, caspase 1, and NF-κB levels. In both a murine model of stroke and in patients, increased IL-1β levels were observed after stroke, and hypothermia treatment was associated with lower IL-1β levels. Furthermore, hypothermia-treated patients showed significant improvement in neurophysiological functional outcome.

**CONCLUSIONS:**

Overall, hypothermia offers clinical benefit, most likely through its effects on the inflammatory response.

## INTRODUCTION

Ischemic stroke is one of the leading causes of mortality worldwide and the number one cause of death in the northern area of China. Inflammatory responses resulting from ischemic stroke have been recognized as a key factor in the pathology of ischemic stroke. Previous studies have shown that the serum interleukin 1β (IL-1β) level is elevated in ischemic stroke patients, indicating activation of the immune system, which is associated with infiltration of immune and inflammatory cells into the central nervous system, possibly mediating neuronal damage in the brain.

Hypothermia is a promising treatment for stroke. Studies of experimental ischemic stroke models have found that the benefits of hypothermia treatment could be the result of a range of biological processes that are modulated by temperature, including reduced oxidative stress, proteolysis, and excitotoxicity (1). More importantly, hypothermia treatment has long been established to reduce the infarction size and cell death due to necrosis and apoptosis (2). Recent studies indicate that therapeutic hypothermia regulates the expression of both pro-inflammatory and anti-inflammatory cytokines, implying a close association between hypothermia and inflammatory responses in the pathogenesis of ischemic stroke (3).

Interleukin 1β (IL-1β), a pro-inflammatory cytokine and a core molecule of inflammasomes, has been found to be associated with neuronal necrosis and apoptosis. Cyclin-dependent kinase 5 (CDK5), in turn, has been reported to mediate the activation of the neuronal inflammasome, accompanied by the expression of core inflammasome molecules, such as caspase 1 (4). Furthermore, hyperactivity of CDK5, caused by the conversion of the CDK5 activator p35 to p25, has been reported to mediate neuronal death in ischemic stroke (5). Therefore, during ischemic stroke, CDK5 may induce activation of the inflammasome, which then leads to neuronal damage. The activation of nuclear transcription factor-κB (NF-κB) has been noted in infarcted cerebral areas during the early stage of ischemic stroke (6). NF-κB is involved in neuronal inflammation after cerebral stroke, but the potential association between CDK5 and NF-κB remains poorly understood.

In this study, we investigated the molecular mechanisms of the inflammatory response in ischemic stroke, particularly the correlation between the levels of CDK5 and various inflammatory molecules, including IL-1β, NF-κB, and caspase 1. Moreover, we further explored the effectiveness of hypothermia as a treatment in a cohort of ischemic stroke patients.

## MATERIALS AND METHODS

The animal experiments were approved by the Animal Ethical Committee of the local hospital.

### Reagents

Neurobasal medium, B27 supplement, high-glucose Dulbecco’s Modified Eagle Medium (DMEM), and fetal bovine serum (FBS) were purchased from Gibco (Grand Island, NY, USA). pcDNA3.0, pcDNA-CDK5, and GFP-p25 plasmids were obtained from Addgene (Cambridge, MA, USA). An NF-κB luciferase reporter plasmid was purchased from Beyotime Institute of Biotechnology (Shanghai, China). Lipofectamine 2000 transfection reagent and Opti-MEMI medium were obtained from Invitrogen (Carlsbad, CA, USA). Etoposide and roscovitine were obtained from Sigma-Aldrich (St. Louis, MO, USA). A luciferase reporter gene assay kit was purchased from Roche (Basel, Switzerland). The 96-well plate used for the luciferase reporter gene test was purchased from Greiner (Lud-wigsburg, Germany). Other cell culture plates were purchased from Corning (Corning, NY, USA). The primary antibody against caspase 1 was obtained from Abcam (Cambridge, MA, USA). Primary antibodies, including anti-CDK5, anti-phosphorylated (p)-CDK5, anti-IL-1β, and anti-β-actin antibodies, were purchased from Santa Cruz Biotechnology (Santa Cruz, CA, USA). Goat anti-rabbit and goat-anti-mouse horseradish peroxidase (HRP)-conjugated secondary antibodies were purchased from Jackson Immuno Research (West Grove, PA, USA).

### Cell culture and transfection

For primary cortical neuronal culture, Sprague-Dawley (SD) rats (prenatal 16-18 days old) were euthanized, and their cortex tissues were collected in D-Hanks solution. The cortex tissues were then digested with trypsin, and the cells were resuspended in neurobasal medium containing B27 supplement and glutamine. The single-cell suspension was then transferred to a 6-well culture plate precoated with poly-D-lysine at a density of 2 × 10^6^ cells/well (in 2 mL culture medium). The cells were incubated at 37°C for 7 days for further experiments. HEK293 cells were cultured in high-glucose DMEM that contained 10% FBS in an incubator at 37°C with 5% carbon dioxide. Transfection was conducted when cells reached 85% confluency according to the manufacturer’s instructions for Lipofectamine 2000. Cells were transfected with the NF-κB luciferase reporter plasmid. Twenty-four hours later, cells were transfected with pcDNA-CDK5 and GFP-p25 plasmids. Control cells were subjected to NF-κB luciferase reporter plasmid transfection followed by transfection with a pcDNA3.0 plasmid. Forty-eight hours after transfection, cells were lysed, and substrates were added according to the manufacturer’s instructions (Roche). The absorption was recorded on a fluorescence microplate, and NF-κB activity was analyzed.

siRNA-CDK5 and siControl were purchased from Genepharma, Shanghai, China. The sequences of siRNAs were as follows: siRNA-CDK5,5′-GUUCAGCCCUCCGGGAGAUTT-3′; SiControl, 5′-GAGACCCTATCCGTGATTA-3′. For RNA silencing, cortical neurons were transfected with siRNAs using Lipofectamine 2000 transfection reagent according to the manufacturer’s instructions. Twenty-four hours after transfection, cells were used for experiments.

### Murine model of ischemic stroke

Male SD rats weighing 350-380 g were used to establish the rodent ischemic stroke model. The animal experiments were approved by the Animal Ethical Committee of the local hospital. The ischemic stroke model was established using the suture-occluded method developed by Longa (7). Three hours after the surgery, rat neurophysiology was evaluated by postural reflex, and unawake or dead animals were excluded from analysis. Rats without autonomous activity or defects in neuronal function were not included in the study. The postural reflex was rated on a three-point scale, and animals that scored ≥1 point were considered ischemic stroke animals.

Animals were assigned to three groups: sham operation control (n=12), ischemic stroke (n=12), and ischemic stroke + hypothermia treatment (n=12). In the ischemic stroke + hypothermia treatment group, rats were treated with a cooling blanket at 3 h after surgery.

### Enzyme-linked immunosorbent assay (ELISA)

Blood samples were collected from animals in the ischemic stroke (n=6) and ischemic stroke + hypothermia treatment (n=6) groups at 6 h after operation. Control animals were fed for 2 days, and blood samples were collected from animals (n=6) under anesthesia. Serum samples were prepared immediately after blood collection. The IL-1β level in serum derived from the three experimental groups was assessed by ELISA.

### Western blotting

Primary cultured neurons were collected and lysed by radioimmunoprecipitation assay (RIPA) lysis buffer. The intracerebral ischemic penumbra was harvested from rats in the ischemic stroke and ischemic stroke + hypothermia treatment groups at 6h after surgery or from control rats. Tissue samples were minced and lysed, and an equal amount of protein was loaded and subjected to gel electrophoresis. Protein samples were then transferred to a polyvinylidene difluoride (PVDF) membrane, which was incubated with primary antibodies against caspase 1 (1:1000 dilution), CDK5 (1:1000), p-CDK5 (1:1000), or β-actin (1:5000) at 4°C overnight. After the membrane was washed with Tris-buffered saline plus Tween-20 (TBS-T) three times, it was further incubated with a secondary antibody (1:20,000) for another 1 h at room temperature. After the membrane was washed, the bands were visualized using the BeyoECL Plus kit according to the manufacturer’s instructions (Beyotime Institute of Biotechnology). The densities of the bands of interest were analyzed by ImageJ software.

### Cohort study of hypothermia treatment

The cohort study included a total of 24 patients with acute cerebral infarction who were treated in the Department of Neurology, the First Affiliated Hospital of Harbin Medical University, Heilongjiang, China, from November 1, 2015 to December 31, 2015. Patients were randomly divided into two groups, including the standard of care group (n=12) and the standard of care + hypothermia treatment group (n=12). In addition, a total of 12 gender- and age-matched healthy controls who underwent physical examination at the First Affiliated Hospital of Harbin Medical University, Heilongjiang, China were recruited. Informed consent forms were collected from all patients. Ethical approval for this study was granted by the local hospital.

Patients who were treated with hypothermia received cooling treatment for 24 h using a focal mild hypothermia therapy device (Harbin, China) wrapped around the head that maintained the tympanic temperature at 33-35°C, which was measured with an OMRON thermometer (Omron Dalian Co., Ltd., China). After therapy, the body temperature was gradually restored to 36.5-37.5°C within 12-20 h, increasing by 1°C every 4-6 h.

Venous blood samples (2 mL) were collected from the patients on the second day after the stroke. Morning fasting venous blood samples (2 mL) were collected from control cases. The serum IL-1β level was assessed by ELISA.

### Statistical analyses

All of the data were presented as the mean ± standard error of the mean (SEM). The statistical analyses were performed with two-tailed Student’s tests. Analyses were carried out by GraphPad Prism5 software. *p-*value of less than 0.05 was considered to be statiscally significant, *p<0.05.

## RESULTS

### Inflammatory molecular signaling pathway in neuronal cultures

We examined the correlation between CDK5 and NF-κB, a molecule known to mediate inflammatory responses. HEK293 cells were transfected with CDK5 together with the CDK5 activator p25 plasmids (CDK5/p25) or pcDNA3.0 plasmid, a negative control, and NF-κB activity was assessed by measurement of the luciferase reporter gene.

**Figure 1:**
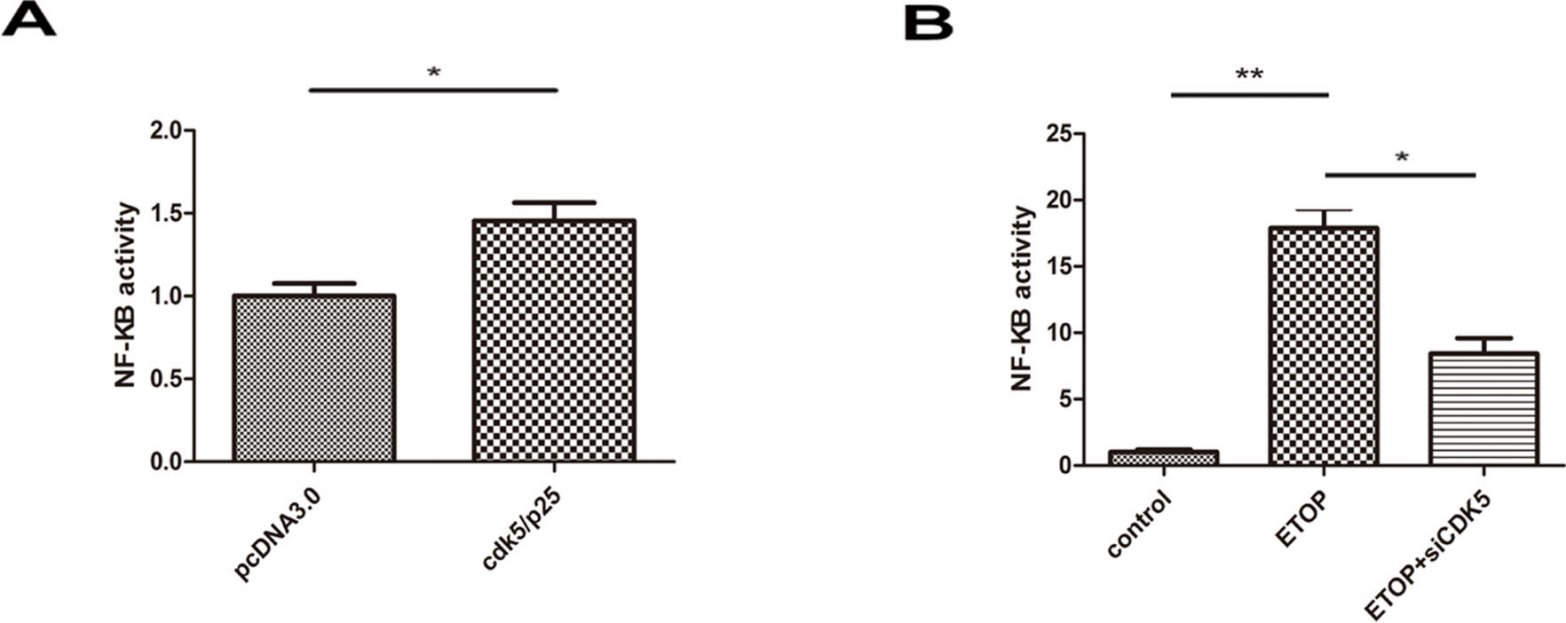
CDK5 activated NF-κB in HEK293 cells and primary cultured cortical neurons. (A) HEK293 cells were transfected with pcDNA3.0 or CDK5 plus p25. (B) Cultured cortical neurons were treated with ETOP to stimulate CDK5 activation or ETOP and siCDK5, and untreated neurons were used as controls. NF-κB activity was measured using a luciferase reporter gene assay. *p<0.05; **p<0.01.

The NF-κB level in cells transfected with CDK5/p25 was significantly higher than that in cells transfected with pcDNA3.0 (Figure 1A). We further examined the correlation between CDK5 and NF-κB in primary cortical neuron cultures. Cultured neurons were treated with etoposide (ETOP) to stimulate CDK5 activation or ETOP and siCDK5, and untreated neurons were used as a control. The activity of NF-κB, as assessed with the luciferase reporter gene assay, was negligible in control cells (Figure 1B). In contrast, ETOP treatment induced substantial NF-κB transcription, which was significantly reduced by cotreatment with siCDK5. Thus, CDK5 may activate NF-κB.

**Figure 2:**
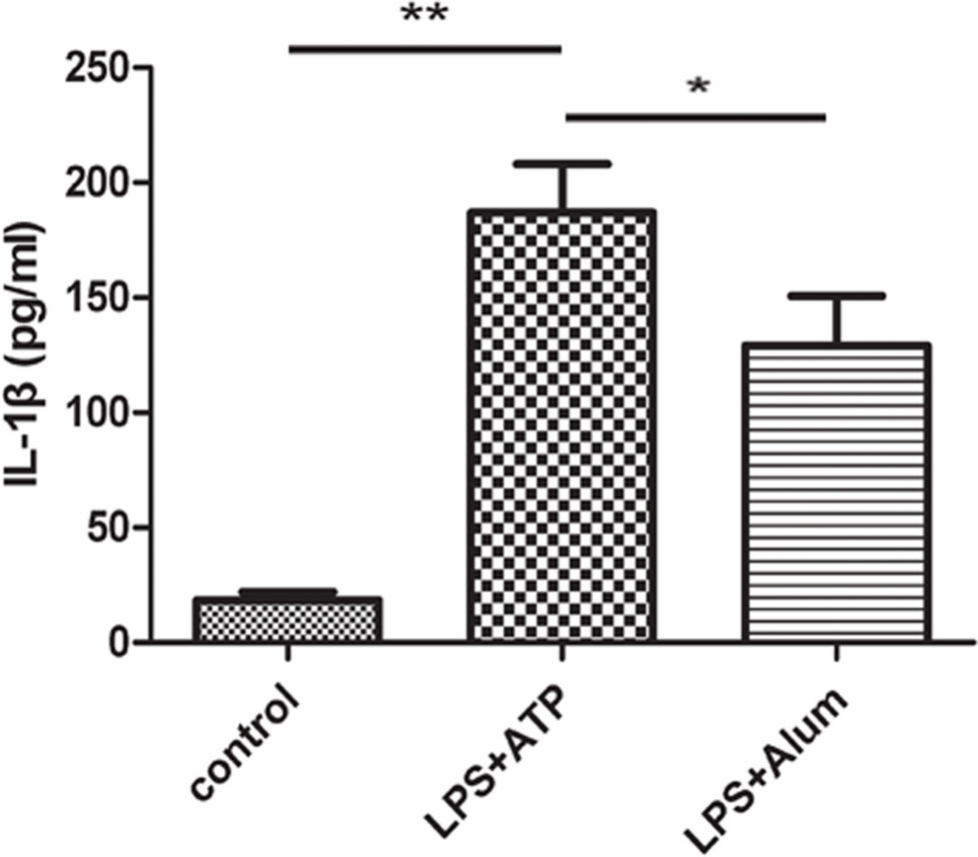
IL-1β was produced in cortical neurons in response to inflammatory stimuli. Cultured primary cortical neurons were treated with LPS and ATP or LPS and alum, and the IL-1β level in the supernatant of the culture was assessed with ELISA. *p<0.05; **p<0.01.

In primary cortical neuronal cultures, inflammatory stimulation was associated with an increase in the IL-1β level. Here, neuronal cultures were treated with lipopolysaccharide (LPS) and adenosine triphosphate (ATP) or LPS and alum, and the IL-1β level in the supernatant of the culture was assessed via ELISA. Cultures treated with either LPS with ATP or LPS with alum had a significantly higher IL-1β level in the supernatant than untreated control cultures (*p*<0.01; Figure 2).

**Figure 3:**
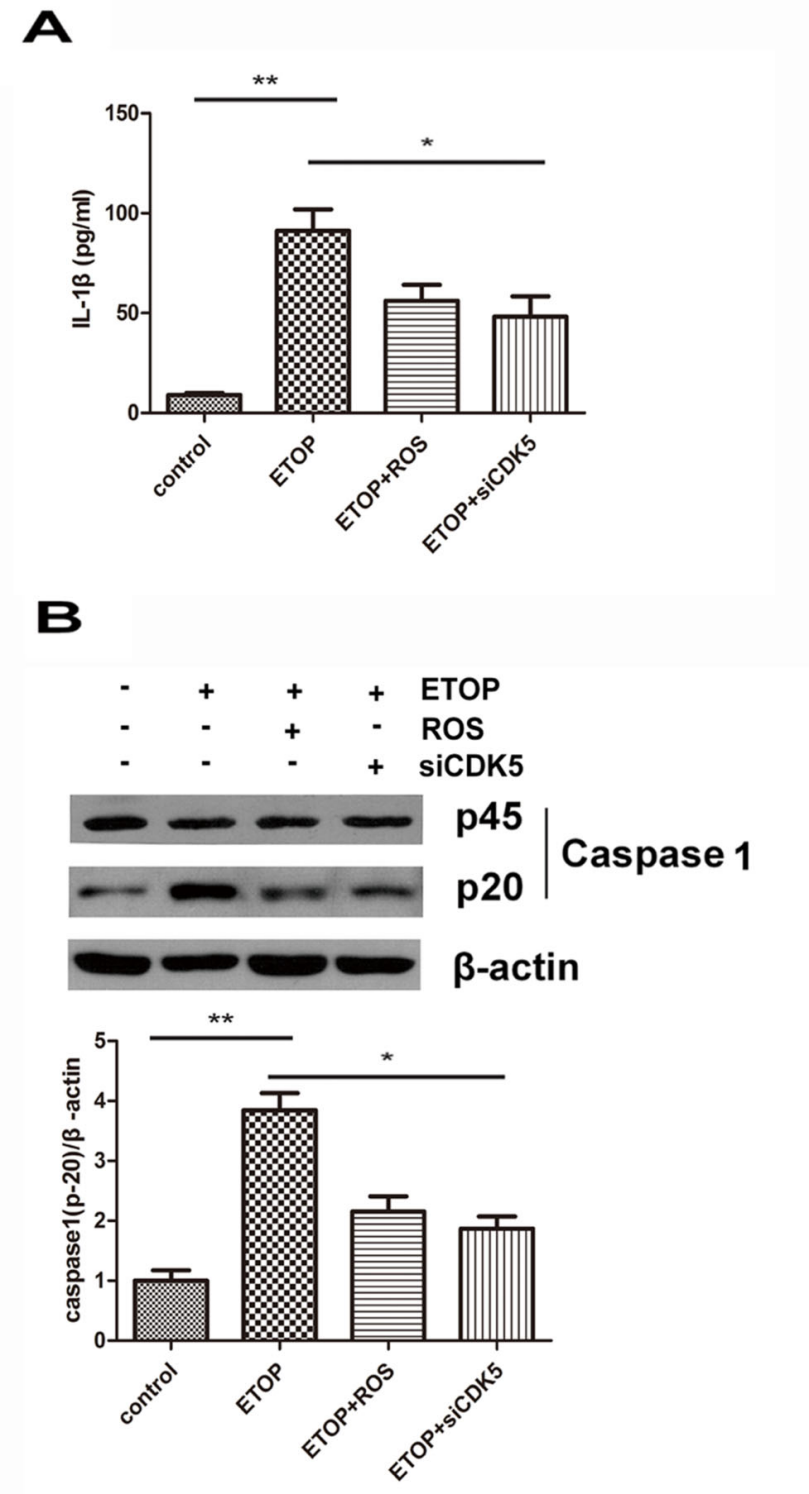
CDK5 provoked the production of IL-1β in cortical neurons. Cultured primary cortical neurons were treated with 10 μM ETOP or 10 μM ETOP plus ROS, a CDK5 inhibitor, for 12h. For RNA interference, cells were pretreated with siCDK5 for 24h, followed by 10 μM ETOP plus siCDK5 incubation for another 12h. Untreated cells were used as the control group. (A) The IL-1β level in the supernatant of the cell culture was assessed by ELISA. (B) The protein expression levels of pro-caspase 1 (p45) and activated caspase 1 (p20) were assessed by Western blotting. *p<0.05; **p<0.01.

Next, we examined the correlation between CDK5 and IL-1β. Cultured primary cortical neurons were treated with ETOP, ETOP plus roscovitine (ROS), a CDK5 inhibitor, and ETOP plus siCDK5. Untreated cells were used as the control group. ELISA revealed that the IL-1β level in the supernatant of the cell culture was significantly higher in the ETOP-treated cells than in the control group (*p*<0.01; Figure 3A). Blocking CDK5 activity with either ROS or siCDK5 significantly reduced the IL-1β level (*p*<0.05). There was no significant difference in the protein expression of pro-caspase 1 (p45) among the three groups as assessed via Western blotting (Figure 3B). The level of activated caspase 1 (p20) in these cells was consistent with the IL-1β level; the p20 subunit level was significantly higher in ETOP-treated cells than in the control group (*p*<0.01), and treatment with either ROS or siCDK5 significantly reduced the IL-1β level (*p*<0.05).

### IL-1β and CDK5 levels in a murine model of ischemic stroke

**Figure 4:**
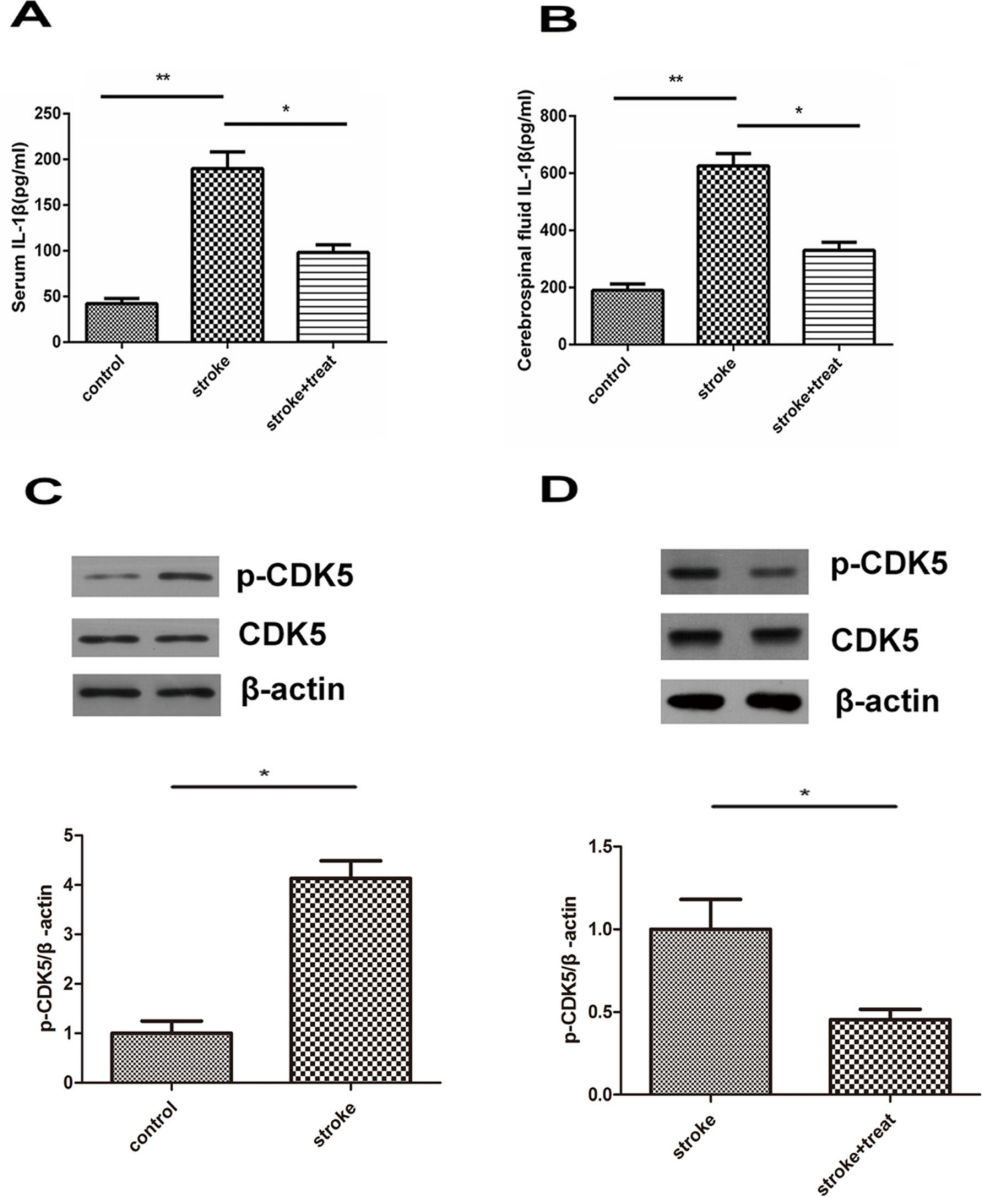
IL-1β and CDK5 levels in a murine model of ischemic stroke. Animals were divided into control, ischemic stroke, or ischemic stroke + hypothermia treatment groups. Serum IL-1β content (A) and IL-1β level in the brain tissue (B) were assessed by ELISA. Six animals from each group were analyzed. (C, D) The protein expression of CDK5 and p-CDK5 in intracerebral ischemic penumbra or control brain tissues was measured by Western blotting. Six animals from each group were analyzed. *p<0.05; **p<0.01.

We next investigated the correlation between hypothermia treatment and IL-1 and CDK5 levels in a rat model of ischemic stroke. Ischemic stroke was surgically induced in rats, and rats in the hypothermia treatment group were cooled immediately after the surgery. Both the serum IL-1β content and IL-1β level in the brain tissue assessed by ELISA were significantly higher in ischemic stroke rats than in rats in the sham surgery group (*p*<0.01; Figure 4A and Figure 4B). Hypothermia treatment significantly reduced the increase in IL-1β (*p*<0.05 compared to ischemic stroke).

In addition, the protein expression levels of CDK5 and p-CDK5 in the intracerebral ischemic penumbra area and normal brain tissues were analyzed. As shown in Figures 4C and 4D, there was no obvious change in the overall expression of CDK5 among the three experimental groups. However, the level of p-CDK5 was significantly upregulated in the intracerebral ischemic penumbra derived from animals with ischemic stroke compared with that in the penumbra from animals who underwent sham surgery (*p*<0.05). Note that treatment with hypothermia greatly reversed the elevation in p-CDK5 in the intracerebral ischemic penumbra in ischemic rats (*p*<0.05).

### Serum IL-1β level in a cohort of ischemic patients treated with hypothermia

This cohort study included 24 patients who had experienced acute ischemic stroke and were randomized into groups treated with hypothermia with standard of care or standard of care alone and 24 healthy volunteers. There was no significant difference in the age between the two cohorts (Table 1; *p*>0.05). Neurophysiological function was assessed using the National Institutes of Health Stroke Scale (NIHSS). At the start of treatment, hypothermia-treated patients scored 13.8±7.8, and the standard of care patients scored 14.2±7.2 (*p*>0.05). After therapy, the decrease in the NIHSS score was 2.6±1.3 in the standard of care group and 3.9±1.6 in the hypothermia-treated patients (*p*<0.05).

**Figure 5:**
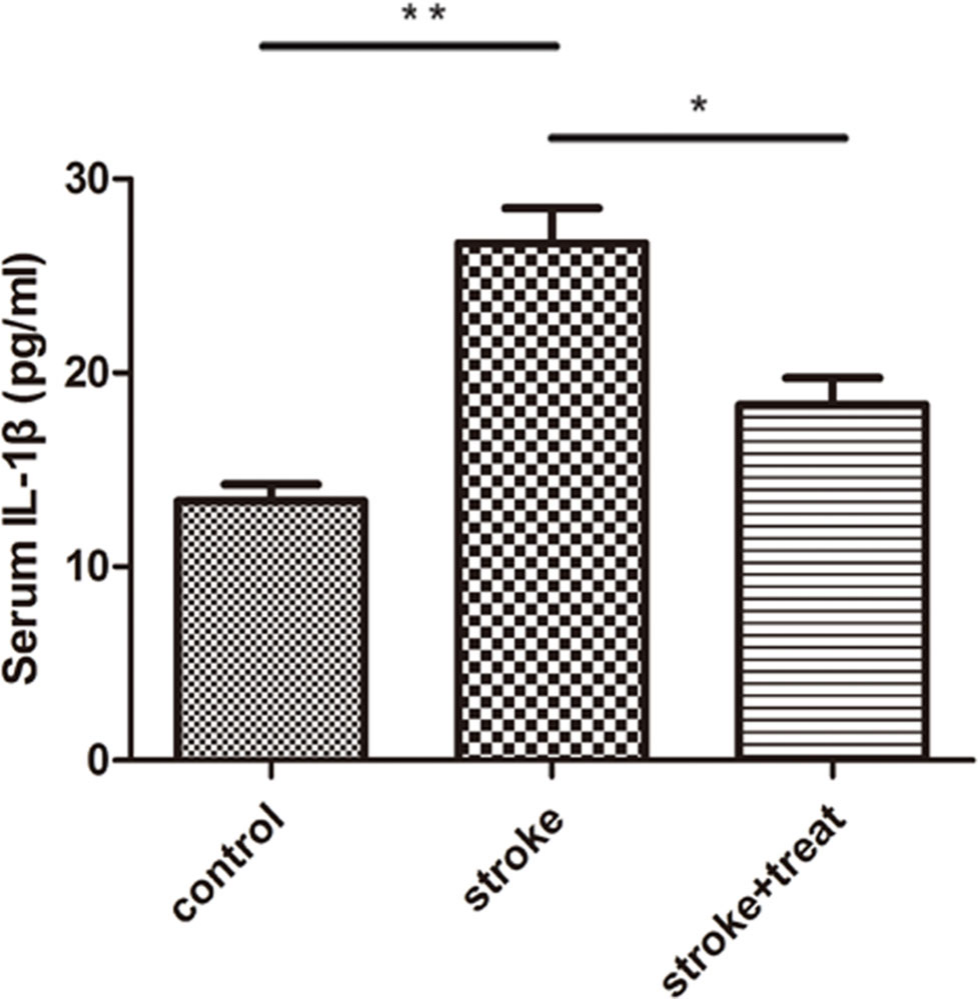
Serum IL-1β levels in a cohort of ischemic patients who were treated with hypothermia. The serum IL-1β level was assessed in all patients (healthy control, n=12; stroke, n=12; stroke + hypothermia treatment, n=12) on the second day after stroke. *p<0.05; **p<0.01.

The serum IL-1β level was assessed in all patients on the second day after the stroke. The IL-1β level was significantly higher in patients with stroke than in healthy volunteers (*p*<0.01; Figure 5). Furthermore, hypothermia-treated patients showed a significantly lower serum IL-1β than patients in the standard of care only group (*p*<0.05).

## DISCUSSION

Hypothermia as a treatment option for ischemic stroke has shown promise. This study examined the molecular mechanism of the inflammatory response and the possible effects of hypothermia on the signaling pathway. We found that in cultured neurons, activation of CDK5 was associated with increased levels of key inflammatory molecules, including IL-1, NF-κB, and caspase 1. These findings are consistent with available studies of inflammatory responses.

A high body temperature is associated with a larger infarct size and poor functional outcome in patients with acute ischemic stroke (8). The effectiveness of therapeutic hypothermia for the management of ischemic stroke has been well documented in several animal studies as well as preclinical trials (9,10). In clinical trials, the use of hypothermia after conventional therapy (e.g., recanalization) has been proven to prevent cerebral edema and improve therapeutic outcomes in patients with acute ischemic stroke (11,12). In rodent studies, therapeutic hypothermia efficiently reduced the infarct size in an ischemic stroke animal model when compared to normothermia (13,14). Although the underlying mechanism by which therapeutic hypothermia protects against cerebral ischemia has not yet been fully clarified, hypothermia is suggested to regulate the inflammatory responses by reducing the expression of pro-inflammatory factors in mice with acute ischemic stroke (15).

The pro-inflammatory cytokine IL-1β plays a crucial role in the pathogenesis of ischemic stroke (16,17). Increased generation of IL-1β in patients with ischemic stroke may induce cell apoptosis, resulting in brain tissue injury in the intracerebral ischemic penumbra area and ultimately enlarging the area of cerebral infarction. In this study, hypothermia treatment efficiently decreased the IL-1β production induced by inflammation in ischemic rodents as well as in patients. Our findings are consistent with a previous study revealing reduced IL-1β expression in stroke mice that received hypothermia therapy (15).

CDK5 is a key molecule that mediates multiple signaling pathways that lead to neuronal loss, including the autophagy process (18,19), apoptosis (20,21), and neuronal death processes (22,23). In particular, CDK5 activity during oxidative stress has been found to contribute to the pathophysiology of various central nervous system disorders (24-26). Our study revealed an increase in CDK5 in animals after ischemic stroke. In addition, CDK5 signaling is closely associated with inflammasome activation; as in primary cultured cortical neurons, activation of the CDK5 pathway in our study promoted the maturation and secretion of IL-1β through activation of caspase 1. In addition, CDK5 activation resulted in increased NF-κB activity in cultured neurons as well as HEK293 cells. Compared to stroke animals, rats receiving hypothermia therapy had a lower p-CDK5 level, indicating that hypothermia suppressed the activation of the CDK5 signaling pathway, consequently decreasing IL-1β generation.

Consistent with previous clinical studies, our study of a small cohort of patients showed clinical benefits of hypothermia treatment. Hypothermia-treated patients showed a better outcome than patients who received only standard of care. This clinical benefit could result from the inhibitory effects of hypothermia on the inflammatory response. Both the brain and serum levels of IL-1β decreased with hypothermia treatment, suggesting that hypothermia at least partially blocks the inflammatory response signaling pathway. However, a recent study found that the combination of mild hypothermia and inhibition of CDK5 showed a trend toward better outcomes (13), indicating that the effects of hypothermia are not limited to suppression of inflammatory responses.

In summary, our present study demonstrates that therapeutic hypothermia exerts neuroprotection in ischemic stroke via a novel mechanism. Therapeutic hypothermia suppresses the abnormal activation of CDK5 in neurons following ischemic stroke, which results in a reduction in NF-κB activity and inhibition of caspase 1, ultimately decreasing the caspase 1-dependent production of IL-1β. In rats with ischemic stroke, therapeutic hypothermia decreases the secretion of the pro-inflammatory cytokine IL-1β, inhibits inflammatory responses, and prevents neuronal apoptosis, thereby providing favorable therapeutic outcomes. Our findings may provide clinical benefits to patients with acute ischemic stroke. Nevertheless, future studies will continue to explore the underlying mechanism of neuroprotection induced by therapeutic hypothermia.

## AUTHOR CONTRIBUTIONS

Sun H designed the study. Ren X analyzed the data. Shen S and Cai J established the rat animal model and carried out immunohistochemistry and electrophoresis studies.

## Figures and Tables

**Table 1:** Demographic and clinical characteristics of study cohorts.

	N	Age (years)	NIHSS score before therapy	Decrease in NIHSS score after therapy
Standard of care	12	62.42±9.91	14.17±7.23	2.75±1.14
Standard of care + hypothermia	12	60.92±11.99	13.83±7.79	3.83±1.27*

* *p*<0.05
